# The Contribution of Mediastinal Transbronchial Nodal Cryobiopsy to Morpho-Histological and Molecular Diagnosis

**DOI:** 10.3390/diagnostics13223476

**Published:** 2023-11-19

**Authors:** Francisco Javier Velasco-Albendea, Juan José Cruz-Rueda, María Jesús Gil-Belmonte, Álvaro Pérez-Rodríguez, Andrés López-Pardo, Beatriz Agredano-Ávila, David Lozano-Paniagua, Bruno José Nievas-Soriano

**Affiliations:** 1 Clinical Management Unit of Pathological Anatomy, Torrecárdenas University Hospital, 04009 Almeria, Spain; fjalbendea@gmail.com (F.J.V.-A.); beatrizagredano@gmail.com (B.A.-Á.); 2 Clinical Management Unit of Pneumology, Torrecárdenas University Hospital, 04009 Almeria, Spain; jucrru@outlook.es (J.J.C.-R.); andreslp9@gmail.com (A.L.-P.); 3 Department of Pathological Anatomy, Hospital Clínico Universitario, 47003 Valladolid, Spain; a.p.rodriguez01@gmail.com; 4 Department of Nursing, Physiotherapy and Medicine, University of Almeria, 04120 Almeria, Spain; brunonievas@ual.es

**Keywords:** cryobiopsy, EBUS-TBNA, mediastinal lymph nodes, lung cancer, biomarkers

## Abstract

(1) Background: endobronchial ultrasound-guided mediastinal transbronchial cryo-node biopsy, previously assisted by fine-needle aspiration, is a novel technique of particular interest in the field of lung cancer diagnosis and is of great utility for extrathoracic tumor metastases, lymphomas, and granulomatous diseases. An integrated histological and molecular diagnosis of small samples implies additional difficulty for the pathologist. Additionally, emerging tumor biomarkers create the need to search for new approaches to better manage the tissue sample; (2) Methods: An analytical observational study of 32 mediastinal node cryobiopsies is carried out in 27 patients (*n* = 27). Statistical analysis using the t-student and Wilcoxon signed-rank tests for paired data is performed with SPSS 26 and R Statistical software. The significance level is established at *p* < 0.05; (3) Results: cryobiopsies were valid for diagnosis in 25 of 27 patients, with a maximum average size of 3.5 ± 0.7 mm. A total of 18 samples (66.67%) were positive for malignancy and 9 (33.33%) were benign. The tumor percentage measured in all neoplastic samples was greater than 30%. The average DNA and RNA extracted in nine non-small cell lung cancer cases was 97.2 ± 22.4 ng/µL and 26.6 ± 4.9 ng/µL, respectively; (4) Conclusions: the sample size obtained from an endobronchial ultrasound-guided mediastinal transbronchial cryo-node biopsy facilitates the morphological and histo-architectural assessment of inflammatory and neoplastic pathology. It optimizes molecular tests in the latter due to more tumor cells, DNA, and RNA.

## 1. Introduction

Different conditions can cause mediastinal lymphadenopathies. Malignant pathology is commonly attributed to lymphomas and metastatic lesions associated with primary thoracic tumors, particularly lung and esophagus, which can spread to the mediastinal nodes [1]. Endobronchial ultrasound-guided transbronchial fine needle aspiration (EBUS-TBNA) and endoscopic ultrasound-guided fine needle aspiration (EUS-FNA) are minimally invasive helpful techniques used to approach the visceral mediastinal compartment. The pathologist’s assistance is crucial in acquiring a rapid on-site evaluation of the sample (ROSE) to optimize and manage the tissue samples beyond the morphological study [1,2,3].

Seventy percent of non-small cell lung cancer (NSCLC) patients are diagnosed in advanced stages without any surgical options, and diagnosis is usually established through cytological samples and small biopsies [4,5]. The proper management of these samples is crucial in making a cytohistological diagnosis of the neoplasm and making them profitable for immunocytochemical/immunohistochemical studies (ICCs/IHCs), fluorescence in situ hybridization (FISH), real-time polymerase chain reaction (RT-PCR), or next-generation sequencing (NGS) [2,3,6,7]. Liquid biopsy is a valuable alternative for molecular testing in lung cancer but requires precise technical requirements and validation studies, particularly regarding circulating tumor RNA [3,8].

According to 2022 guidelines (SEAP/SEOM) for NSCLC, it is recommended to assess the mutational status of *EGFR*, *BRAF*, *KRAS*, and *MET*, as well as *ALK*, *ROS1*, *NTRK*, and *RET* translocations and PD-L1 expression levels. If NGS is available, the detection of emerging immunomarkers such as *HER2*, *MSI*, *STK11*, *KEAP1*, or TMB should be considered. Therefore, pathologists need to handle samples obtained from patients with lung cancer in a specialized, systematic, and directed way to make them profitable for diagnosis while preserving tissue for molecular determinations [6].

Cryobiopsy in interstitial lung disease is a widespread multidisciplinary procedure in the pathologist’s diagnostic routine [9]. Endobronchial ultrasound-guided transbronchial mediastinal lymph node cryobiopsy (EBUS-TBCNB), previously assisted by EBUS-TBNA, is a valuable technique because of the larger tissue sample size obtained. EBUS-TBCNB complements conventional approach techniques used for sampling NSCLC patients with the intention of staging, categorizing tumor histological type, and responding to the molecular requirements in lung cancer. It also facilitates the diagnosis of other inflammatory and neoplastic pathologies involving the mediastinal lymph nodes [10,11,12,13,14,15,16,17,18,19]. However, no studies have analyzed mediastinal lymph node cryobiopsy from the pathologist’s perspective.

Therefore, the study’s main objective was to obtain data on the overall diagnostic with the EBUS-TBCNB technique associated with EBUS-TBNA. We further sought to assess the relevance of the pathologist’s assistance in decision-making during ROSE, to check the efficacy of this type of biopsy for molecular testing by quantifying the amount of DNA and RNA in comparison with the cell blocks obtained in EBUS-TBNA, and to evaluate the safety of the technique.

## 2. Materials and Methods

A prospective, descriptive, observational, and analytical study was conducted from April 2022 to April 2023 on patients who underwent EBUS-TBCNB at the level 1 Torrecárdenas University Hospital in Almería, Spain. This hospital covers a health area of over 730,000 inhabitants. All the patients had previously undergone computed axial or positron emission tomography/computed tomography studies.

The selection criteria were patients with accessible stations through EBUS-TBNA, patients with mediastinal lymph node pathology (inflammatory or tumor) requiring histological confirmation diagnosis, NSCLC patients with mediastinal lymph node pathology requiring staging or biomarker studies, patients with metastatic mediastinal lymph node pathology related to other primary neoplasms other than lung that benefited from molecular studies, patients with mediastinal lymph node pathology with suspected lymphoma, adults over 18 years of age, and those with no contraindications for sedation or general anesthesia.

### 2.1. Technical EBUS-TBCNB Procedure

The procedure was performed under sedation with a level of consciousness measured using the bispectral index (BIS) around 70–60. Using EBUS-TBNA (Olympus^®^ BF-UC180F, Tokyo, Japan), three punctures were performed with a needle size of either 22 gauge (SonoTip^®^TopGain: Medi-Globe, Rohrdorf, Germany), 19 gauge (ViziShot 2 FLEX: Olympus, Tokyo, Japan), or 21 gauge (ViziShot 2: Olympus, Tokyo, Japan). The pathologist could visualize cytological extensions fixed in alcohol (96°) and stained with Papanicolaou in each puncture, indicating whether there was a representative lymph node component and whether the diagnostic suspicion was benign or malignant. Material from the different punctures was processed in fixative (ThinPrep^®^ CytoLyt Solution, Hologic Inc., Marlborough, MA, USA) for liquid-based cytology and, if applicable, flow cytometry in phosphate-buffered saline (PBS). The tissue casts or cylinders obtained were deposited in 10% diluted formaldehyde to generate cell blocks and processed as small biopsies.

**Figure 1:**
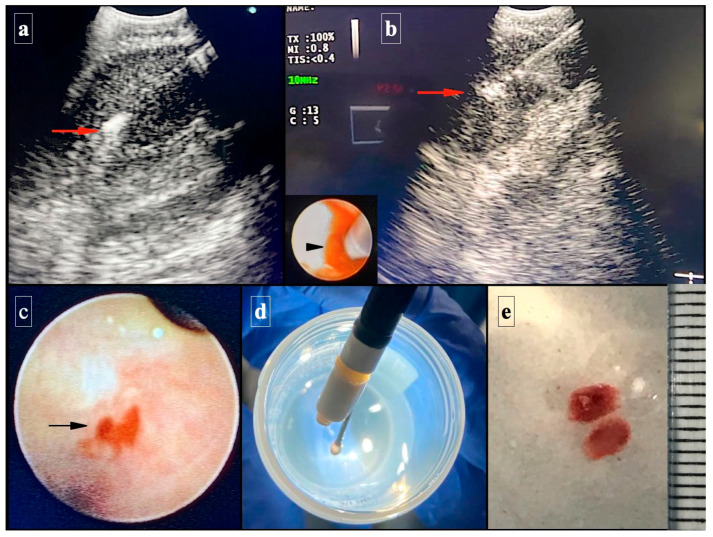
Images of the cryoprobe tip inside the lymph node (red arrows) in an endobronchial ultrasound-guided transbronchial mediastinal lymph node cryobiopsy procedure (EBUS-TBCNB). See the detail in the image of the circle of the cryoprobe entry through the preceding linear EBUS working channel (arrowhead) (**a**,**b**). The bleed point (black arrow) marks the puncture site of the linear EBUS and cryoprobe approach (**c**). Introduction of the cryoprobe with the sample attached to its tip into the buffer solution (**d**). Samples were collected using cryobiopsy (**e**).

After the final puncture, a 1.1 mm cryoprobe (Erbecryo 20402-401, Tubingen, Germany) was inserted through the working channel of the linear echo-bronchoscope and moved towards the puncture site through the hole created by the needle. An ultrasound image was used to confirm the position of the cryoprobe within the lymph node; then, the cryoprobe was frozen for 3–4 s. The ultrasound bronchoscope was then removed with the obtained biopsy attached to the tip of the cryoprobe. The cryobiopsies were placed in a phosphate buffer solution (0.15–0.2% NaH_2_PO_4_, 0.7–0.8% Na_2_HPO_4_, and deionized water). Then, purified 12% formaldehyde was added, generating a 10% buffered formalin (Figure 1).

**Figure 2:**
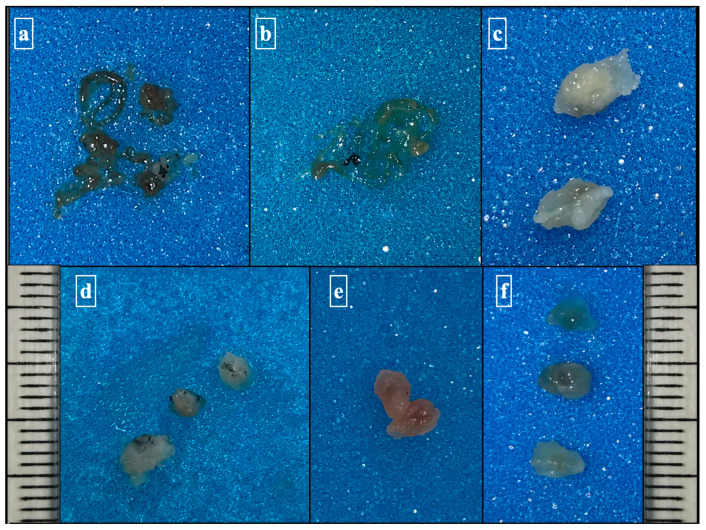
Chordonal material and small irregular fragments were obtained as cellblocks in linear EBUS (**a**,**b**). Macroscopic images of cryobiopsies composed of fragments ranging from 3 mm to 4 mm (**c**–**f**).

The pathologist completed the deferred study of the cytological smears generated with the liquid cytology, cellblocks, and lymph node cryobiopsies (Figure 2). The same pathologist who checked the cytological material and cell blocks of the EBUS-TBNA diagnosed EBUS-TBCNB.

### 2.2. Molecular Study

The molecular biomarkers (*EGFR*, *BRAF*, *KRAS*, *MET*, *ALK*, *ROS1*, *NTRK* y *RET*) were performed using RT-PCR (MagCore^®^ Plus II, RBC Bioscience, Taipei, Taiwan and EasyPGX^®^ qPCR instrument 96, Diatech Pharmacogenetics S.R.L. Jesi AN, Italy). DNA and RNA purity and concentration were assessed using UV absorbance at 260 and 280 nm, employing the NanoDrop 2000 (ThermoFisher Inc., Waltham, MA, USA). The manufacturer’s instructions were followed for assessing DNA purity, where the ratio of absorbance at 260 and 280 nm was considered sufficient if it was between 1.8 for DNA and 2.0 for RNA. The UV absorbance at a ratio of 260/230 nm was utilized as a secondary criterion. A ratio of 2.0–2.2 was deemed sufficient for ensuring sample purity. In all EBUS-TBCNB cases where RT-PCR was performed, extracted DNA and RNA quality were bounded in those purity ranges. To ensure accurate amplification, we confirmed that the cut-off point for DNA extraction was 20 ng/µL and, for RNA extraction, it was 15 ng/µL. The expression level of PD-L1 was assessed using IHC (clone 22C3, DAKO). The BRAFV600 mutational study was conducted using RT-PCR “Cobas^®^ BRAF/NRAS FFPET Mutation Test LSR” Cobas BRAF v2—BRAF V600 mutated [E/E2/D/R/K(M)] (Roche Diagnostic, Basilea, Switzerland). NGS was performed with “Panel Archer FusionPlex Sarcoma” (sequencing trial using Miseq System Illumina, San Diego, California, USA analyzed with Archer Analysis v5.0). Detection of t(X;18) was performed using break-apart FISH.

### 2.3. Statistical Analysis

A descriptive study was conducted to examine the characteristics of the population and technical parameters of the biopsy, namely, needle size, sample size, number of fragments, diagnoses made, percentage of tumor cells, and DNA and RNA quantification. Measures of central tendency, including mean and standard deviation, were used for quantitative variables, while frequencies and proportions were utilized for qualitative variables. Student’s *t*-test or Wilcoxon signed-rank test was used to establish whether there were statistical differences in DNA and RNA quantification between the two techniques. The Kolmogorov–Smirnov test was performed to assess the normality of the continuous variables. Analyses were performed with R Statistical Software (v4.1.2; R Core Team 2021) and SPSS version 26 (IBM Inc., Armonk, NY, USA).

### 2.4. Ethical Aspects

The study was conducted following the Helsinki Declaration guidelines. The participants were given informed consent, and their data were pseudonymized for biomedical research purposes. The study has been approved by the provincial research ethics committee of Almería (code: 25/2023).

## 3. Results

The study analyzed 27 patients and conducted 32 mediastinal lymph node cryobiopsies, and 29 were valid for diagnosis. The remaining three were limited to the lymph node capsule corresponding to perinodal soft tissues, with two biopsies belonging to the same patient. All lymph nodes were larger than 10 mm.

Table 1 summarizes the patients’ demographic data, smoking habits, and ROSE attendance. Two stations were biopsied in five patients, and one was sampled in 22. The stations that were sampled included G7 (23), 11R (4), 4R (2), 11L (1), 4L (1), and 10L (1).

Table 2 provides information regarding the technical parameters of the biopsy.

Eighteen (66.67%) patients had malignant neoplasms: twelve had primary lung tumors and six had extrathoracic malignancies. The remaining nine patients (33.33%) were diagnosed with inflammatory conditions (two patients with invalid cryobiopsies were classified as benign based on the EBUS-TBNA). All malignant neoplasms had a tumor cell percentage exceeding 30%. The histological diagnoses in the cryobiopsies and the diagnostic categories assigned in the EBUS-TBNA [20] are listed in Table 3.

**Figure 3:**
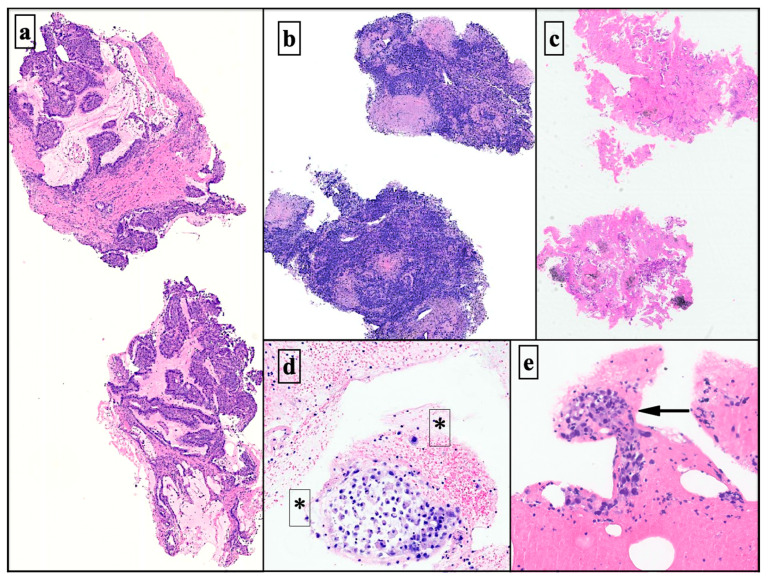
Slight magnification histological details of mediastinal lymph node cryobiopsy specimens almost entirely replaced by lung adenocarcinoma metastases (H&E ×2) (**a**). Representative lymph node fragments were obtained using cryobiopsy with the granulomatous histoarchitectural disorder (H&E ×2) (**b**). Microscopic view of lymph node cryobiopsies with tumor characteristics (H&E ×1) (**c**). Histology of linear EBUS cell blocks with neoplastic non-small cell lung carcinoma (NSCLC). Cells with no defined architectural pattern in a haematic background (asterisks and black arrow) (H&E ×5) (**d**,**e**). H&E: Hematoxylin–Eosin stain.

**Figure 4:**
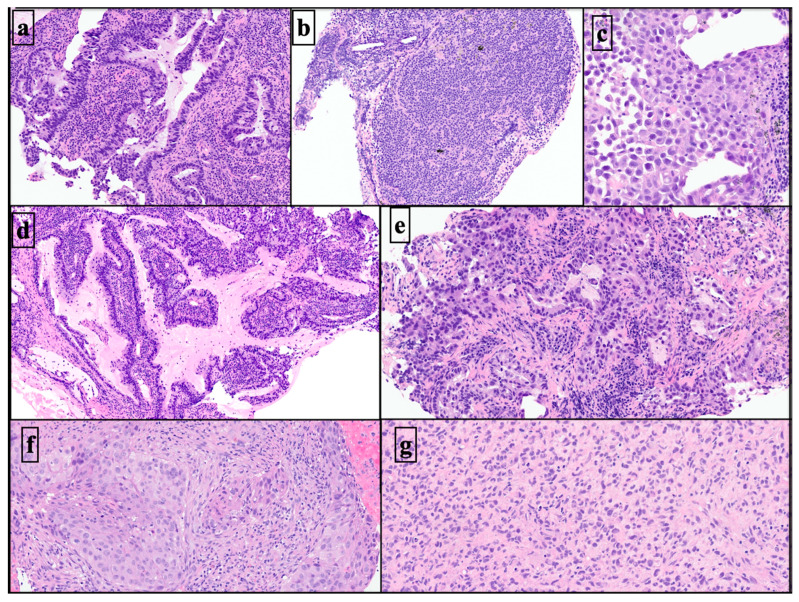
Histoarchitectural patterns in neoplastic cryobiopsy specimens. Acinar adenocarcinoma of the lung with well-defined glands replacing part of the lymphoid nodal tissue (H&E ×5) (**a**). Small cell lymphocytic lymphoma with diffuse pattern (H&E ×4) (**b**). Nodal metastasis from melanoma is composed of discohesive epithelioid cells with marked anaplasia and diffuse growth (H&E ×10) (**c**). Metastasis from adenocarcinoma of the lung with a papillary architectural pattern (H&E ×5) (**d**). Metastasis from acinar adenocarcinoma with irregular and fused glands with associated fibrodesmoplastic stroma (H&E ×7.5) (**e**). Pulmonary squamous cell carcinoma forms well-defined polygonal cell nests with dyskeratosis (H&E ×8) (**f**). Poorly differentiated sarcomatous neoplasm with diffuse pattern (H&E ×7.5) (**g**). H&E: Hematoxylin–Eosin stain.

**Figure 5:**
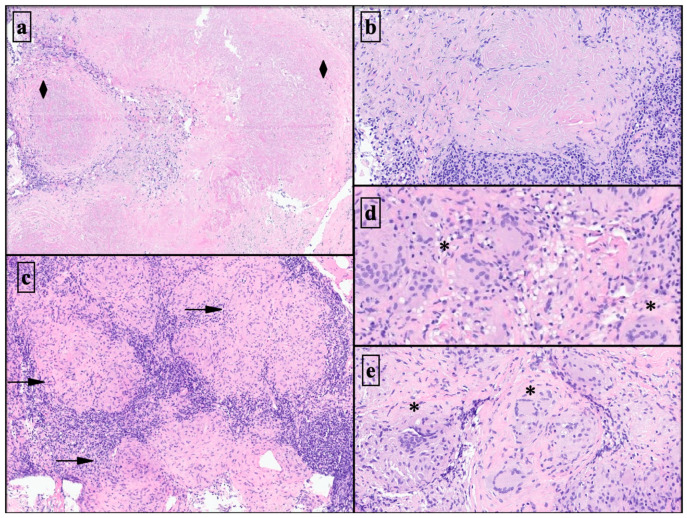
Histoarchitectural patterns in non-tumor cryobiopsy samples. Microscopic view of the nodular granulomatous lesion (black diamonds) in a patient with silicosis (H&E ×4) (**a**). Details of silicoid granulomatous nodule with lamellar fibrosis (H&E ×5) (**b**). The well-defined architecture of granulomatous sarcoid pattern in lymph node (black arrows) (H&E ×5) (**c**). Non-necrotizing granulomatous inflammation in pneumoconiosis with multinucleated giant cells (asterisks) is easily identified after cryobiopsy (H&E ×10) (**d**,**e**). H&E: Hematoxylin–Eosin stain.

The histo-architectural characterization in the 29 cryobiopsies had higher definition and resolution than in the cell blocks produced through the associated EBUS-TBNAs (Figure 3, Figure 4 and Figure 5).

Out of the 12 cryobiopsies conducted for lung malignancies, nine NSCLC (eight adenocarcinomas and one large cell neuroendocrine carcinoma) were valid for RT-PCR and PD-L1 IHC testing, leaving the EBUS-TBNA material for IHC determinations (p40 and TTF1). Only PD-L1 was determined in one squamous NSCLC, as age did not require other biomarkers to be tested. No ancillary biomarker determination was tested in the two small-cell neuroendocrine carcinomas.

**Figure 6:**
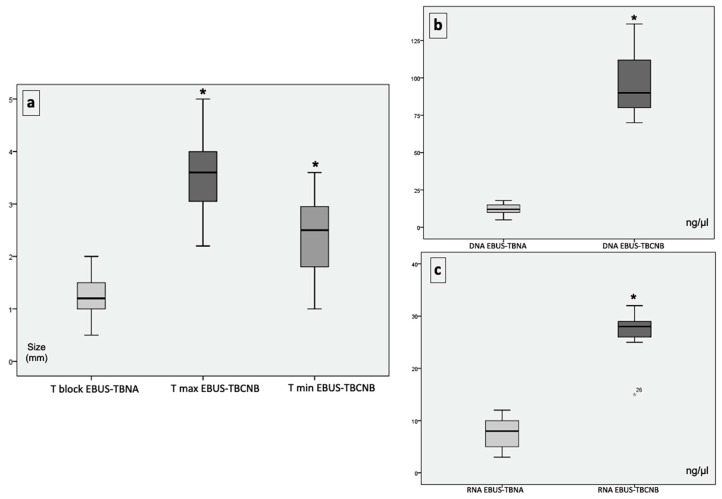
Graphical representation of sample size (mm). T block (cellblock size in EBUS-TBNA). T max (maximum cryobiopsy size). T min (minimum cryobiopsy size). Statistically significant differences (asterisks) versus cell block size in EBUS-TBNA (*p* < 0.05) (**a**). Graphical representation of the amount of DNA and RNA (ng/µL) extracted in EBUS-TBNA cellblocks and cryobiopsies. Statistically significant differences (asterisks) versus the amount of DNA/RNA in EBUS-TBNA cellblocks (*p* < 0.05) (**b**,**c**).

The associated bivariate analysis in Figure 6 showed that the amounts of DNA and RNA extracted from the cryobiopsies of eight adenocarcinomas (29.63%) and one large cell neuroendocrine carcinoma (3.70%) were significantly higher compared to the associated EBUS-TBNA cellblocks (97.2 ± 22.4 ng/µL and 26.6 ± 4, 9 ng/µL versus 11.8 ± 4.3 ng/µL and 7.7 ± 3.6 ng/µL, respectively).

Molecular results were as follows: one adenocarcinoma with *EGFR* mutation (L858R, exon 21) with remaining native biomarkers; one adenocarcinoma with *NTRK* mutation (NTRK3ex15) with remaining native biomarkers; six adenocarcinomas and one large cell neuroendocrine carcinoma with native *EGFR*, *BRAF*, *ALK*, *ROS1*, *RET*, *MET*, and *NTRK*. PD-L1 immunohistochemical expression levels in the 10 NSCLCs, including one squamous cell carcinoma, were 80% (1), 20% (1), 15% (1), 5% (3), and <1% (4). Other molecular results included three melanomas: two with a BRAF V600 mutation and one with native BRAF V600 and one sarcoma. NGS was targeted to simultaneously detect and identify the fusions of 26 genes (*ALK NM*, *CAMTA1*, *CCNB3*, *CIC*, *EPC1*, *EWSR1*, *FOXO1*, *FUS*, *GLI1*, *HMGA2*, *JAZF1*, *MEAF6*, *MKL2*, *NCOA2*, *NTRK3*, *PDGFB*, *PLAG1*, *ROS1*, *SS18*, *STAT6*, *TAF15*, *TCF12*, *TFE3*, *USP6*, and *YWHAE*) associated with soft tissue cancers from negative patient RNA. SYT FISH (t(X;18) (p11;q11) translocation was used for synovial sarcoma along with a break-apart probe (negative). Cryobiopsies performed on small cell lymphocytic lymphoma and urothelial carcinoma metastasis were combined with EBUS-TBNA samples for flow cytometry (only for lymphoma cases) and IHC (in both cases). EBUS-TBCNB samples in all non-tumor lesions allowed for the planning of IHCs and histochemical (HCs) studies. The needle size did not affect the measurements. No complications were detected in the 32 EBUS-TBCNBs.

## 4. Discussion

This study shows the results of 32 cryobiopsies performed in different mediastinal lymph node stations in 27 patients for one year, highlighting the pathologist’s assistance with interventional pulmonologists during EBUS-TBNA with EBUS-TBCNB procedures and emphasizing the relevance of adequate management and the optimization of the samples obtained in both methods. In lung cancer diagnosis, the pathologist usually handles cytology or small biopsies wherein the tumor fraction and percentage of viable tumor components are limited for a comprehensive morphophysiological and molecular diagnosis [2,3,4,6]. Advances in lung cancer treatment entail making these tissues as profitable as possible for the pathologist. Depending on the laboratory’s technology, the professionals’ experience, and the pathologist’s participation in ROSE, there are differences in results between institutions [2,3,4,5].

Quality samples become essential for obtaining histological and molecular guarantees in cases of lung cancer [4,5]. In the approach to identify and examine mediastinal lymph nodes in lung cancer, the metastatic processes of extrathoracic tumors, lymphomas, or granulomatous diseases, EBUS-TBNA has shown excellent cost-effectiveness in hospitals with experienced interventional teams, especially when optimizing with ROSE [2,3,6,7,20,21]. However, sometimes, sample limitation hinders the study of large IHC panels or disables the determination of biomarkers requested by oncologists. In these cases, EBUS-TBCNB becomes a valuable tool for diagnostic support.

Transbronchial cryobiopsy samples, due to their larger size, allow the pathologist to recognize the histoarchitecture and better interpret histological patterns in an adequate clinical-radiological context. Additionally, in malignant neoplasms, they help obtain a percentage of tumor cells valid for molecular tests [9,17].

Our EBUS-TBCNB series is one of the largest published [10,11,13]. The results of this technique initiated by Ariza-Prota and collaborators [13] are promising, and our work offers a novel perspective from the pathologist that has yet to be performed [10,11,12,13,14,16,17,19]. Our experience in EBUS-TBNA with ROSE exceeds 300 cases and has facilitated the implementation of EBUS-TBCNB; the cryoprobe tip did not reach the interior of the lymph node in only two patients. When the pathologist participates in EBUS-TBCNB, the benefits of ROSE in the EBUS-TBNA that precedes are added, namely, clinical-radiological and pathological contextualization, information directed to the pulmonologists during the performance of the successive passes, greater certainty of adequacy, and increased representativeness of the sample for complementary studies [3,6].

Depending on the diagnostic suspicion, the need for complementary studies for molecular studies is assessed, especially in the second approach: the pathologist assesses the validity of the EBUS-TBNA samples. Together with the pulmonologists, they decide whether EBUS-TBCNB favors a more precise and comprehensive diagnosis. In our series, the same pathologist managed the EBUS-TBNA study and the cryobiopsy fragments.

In 22 patients, the pathologist assisted in ROSE, and cryobiopsy was decided when the possible diagnostic limitations were verified to determine if they exclusively handled the material obtained in EBUS-TBNA. Within the five cryobiopsies without previous ROSE, two had a high suspicion of small cell neuroendocrine carcinoma, wherein the molecular needs were not so demanding, and three cases already had a previous diagnosis (two NSCLC and one inflammatory), directing the EBUS-TBCNB for molecular determinations and histological diagnosis.

EBUS-TBNA is a minimally invasive technique that achieves good results and diagnostic yield in lung cancer and malignant neoplasms in hilar and mediastinal nodes > 95% and 80% of the samples that are suitable for IHC and molecular testing [15,18,21]. For the pathologist, having EBUS-TBCNB samples available offers a solution for those cases where EBUS-TBNA is insufficient or limited for diagnosis, with or without ROSE assistance. It opens options for emerging biomarkers in NSCLC. The molecular yield of our series is 100% when performed in the 13 cases that required it (nine NSCLCs, three melanomas, and one sarcoma).

The diversity of samples combining EBUS-TBNA and EBUS-TBCNB allows the pathologist to manage and make the material profitable in a targeted manner. In lung cancer, it facilitates the application of the recommended molecular test guidelines. In other pathologies, it favors the planning of complementary studies [6]. These decisions are usually complicated due to the limitations of some EBUS-TBNA samples.

Cryobiopsies in cases of granulomatous diseases versus EBUS-TBNA cellblocks offer a more complete architectural morphological histological study. In neoplasms, morphologic and IHC diagnosis could be approximated on EBUS-TBNA cellblocks and cytology samples. EBUS-TBCNB better defined histologic patterns, secured tissue for expanded IHC panels, and warranted molecular studies. High-grade sarcoma was diagnosed using EBUS-TBCNB; EBUS-TBNA oriented toward mesenchymal neoplasm, category VI [20]. Using IHC, the use of cryobiopsy was decisive in ruling out the existence of neoplastic cells in one of the granulomatous lymphadenitis of a patient treated for testicular seminoma, and the architectural pattern was established in small cell lymphocytic lymphoma. Our results support the value of EBUS-TBCNB as a complementary procedure rather than an alternative to EBUS-TBNA.

EBUS-TBCNB did not generate any relevant complications, which aligns with the literature [10,11,12,13,14,16,17,19]. Regardless, minor bleeding (less than 10 mL of blood) resulting from the previous puncture with the aspiration needle used in EBUS-TBNA has been detected in most cases. However, no intervention was necessary to address it. It is also worth noting that, during subsequent EBUS-TBCNB procedures, there was no increase in bleeding as the same working channel was utilized. Other complications, such as pneumothorax, mediastinitis, pneumomediastinum, and hemomediastinum, have been described in a small percentage of cases [11,19,22]. The times employed ranged from 10 to 15 min in addition to the time spent in EBUS-TBNA, and none of the 18 tumor cases required a re-biopsy.

Applying cold with the cryoprobe (3–4 s) probably poses a challenge in preanalytical DNA and RNA extraction and analysis. However, the biomarker determinations in our series, which were based on RT-PCR and NGS, have not been compromised, as in previously published series [7,10,11,12,13,14,15,17,18,19]. The percentage of tumor cells recommended for NGS is 20–30% and 5% for RT-PCR [6]. The tumor node cryobiopsy samples in our series harbored greater than 30%, and the amounts of DNA and RNA extracted were high. The quantification of DNA and RNA (ng/µL) in cryobiopsy samples has not been previously published. The results of the nine NSCLCs demonstrate an amount of DNA and RNA much higher than that extracted in the cell blocks in EBUS-TBNA. Although validation in more extensive cases is required, this may be an alternative for studying biomarkers in NSCLC.

It is also important to consider that EBUS-TBNA and EBUS-TBCNB obtain samples from the same patient; therefore, they complement each other. Cryobiopsies, being larger and having a higher percentage of tumors (>30%), are especially valuable for molecular studies. If the percentage of tumors exceeds 30% in cryobiopsies, it makes them valid even for NGS, which is sometimes difficult to ensure in cell blocks.

On the one hand, the most relevant limitation of this study is the potential selection bias. The available data guarantee no clear confounding bias if results are extrapolated to populations with inclusion criteria superimposable to those cited. On the other hand, this is the first study to compare EBUS-TBCNB versus EBUS-TBNA from the pathologist’s point of view. It is worth noting that the findings presented in this report should be approached with caution due to the limited number of available samples, mainly due to the novelty of the technique. Nevertheless, given that this study has demonstrated promising results, it would be interesting for future research to compare their findings with ours to further validate the techniques used.

## 5. Conclusions

EBUS-TBCNB can be a complementary technique to EBUS-TBNA. A larger sample size allows a histo-architectural reading that facilitates the pathologist’s diagnosis of mediastinal neoplastic and inflammatory nodal disease. A higher percentage of tumor cells in cryobiopsy with high-quality DNA and increased RNA quantities ensures greater cost-effectiveness for molecular studies, adding value to NSCLC that can be used to address emerging biomarkers. This procedure is fast, minimally invasive, and avoids the need for re-biopsies.

The pathologist’s management of samples in situ in EBUS-TBNA facilitates the decision of whether a patient benefits from EBUS-TBCNB. Our results, obtained from the pathologist’s perspective, something that has been previously undocumented, support the incorporation and standardization of this technique to optimize morphologic and molecular diagnoses in cases of mediastinal adenopathies. Future studies could compare their findings with ours.

## Figures and Tables

**Table 1:** Patient characteristics and assistance in ROSE.

		*N* (%)/Mean ± SD
Gender	Male	20 (74.07%)
	Female	7 (25.93%)
	Total	27 (100%)
Age		56.1 ± 14.6
Tobacco	Smoker	6 (22.22%)
	Non-smoker	11 (40.74%)
	Former smoker	10 (37.04%)
	Total	27 (100%)
ROSE ^1^	Yes	22 (81.48%)
	No	5 (18.52%)
	Total	27 (100%)

^1^ ROSE: Rapid on-site evaluation.

**Table 2:** Needle size used in linear EBUS and the number and average of the sample size.

		*N* (%)
Needle size	19 G	4 (14.81%)
	21 G	4 (14.81%)
	22 G	19 (70.37%)
	Total	27 (100%)
Number of cryobiopsy fragments		3.4 ± 1.4
EBUS-TBCNB maximum fragment sizes (mm)		3.5 ± 0.7
EBUS-TBCNB minimum fragment sizes (mm)		2.3 ± 0.7
EBUS-TBNA cell block sizes (mm)		1.2 ± 0.5

Linear EBUS: linear endobronchial ultrasound-guided.

**Table 3:** Diagnoses performed in EBUS-TBNA and EBUS-TBCNB.

		*N* (%)
Diagnostic categories in EBUS-TBNA ^1^	I	0 (0%)
	II	9 (33.33%)
	III	0 (0%)
	IV	0 (0%)
	V	0 (0%)
	VI	18 (66.67%)
	Total	27 (100%)
Histological diagnoses in EBUS-TBCNB	Adenocarcinoma metastases	8 (29.63%)
	Squamous cell carcinoma metastases	1 (3.70%)
	Small cell neuroendocrine carcinoma metastases	2 (7.41%)
	Large cell neuroendocrine carcinoma metastases	1 (3.70%)
	Melanoma metastasis	3 (11.11%)
	Lymphoma	1 (3.70%)
	Sarcoma	1 (3.70%)
	Urothelial carcinoma metastases	1 (3.70%)
	Granulomatous lymphadenitis *	5 (18.52%)
	Reactive lymphadenitis **	2 (7.41%)
	Not valid for diagnosis	2 (7.41%)
	Total	27 (100%)
Malignant neoplastic processes	Yes	18 (66.67%)
	No	9 (33.33%)
	Total	27 (100%)
Lung primary origin	Yes	12 (66.67%)
	No	6 (33.33%)
	Total	18 (100%)

^1^ The Papanicolaou Society of Cytopathology System for Reporting Respiratory Cytology. * Granulomatous lymphadenitis diagnosed in EBUS-TBCNB: sarcoidosis (*n* = 1) and pneumoconiosis (*n* = 4). ** Reactive lymphadenitis diagnosed in EBUS-TBCNB: acute abscessed lymphadenitis (*n* = 1) and pseudogranulomatous lymphadenitis associated with chemotherapy-induced changes in a testicular seminoma (*n* = 1).

## Data Availability

The data presented in this study are available on request from the corresponding author.
